# Safety and feasibility of left atrial appendage inversion in swine: A proof-of-concept study for potential therapy to prevent embolic stroke

**DOI:** 10.3389/fbioe.2023.1011121

**Published:** 2023-02-16

**Authors:** Yanmin Wang, Mengjun Wang, Xiaomei Guo, Ling Han, Ghassan Kassab

**Affiliations:** ^1^ California Medical Innovations Institute, San Diego, CA, United States; ^2^ 3DT Holdings, LCC, San Diego, CA, United States

**Keywords:** atrial fibrillation, stroke, atrial appendage, echocardiogram, thoracotomy

## Abstract

**Objective:** Left atrial appendage (LAA) occlusion or exclusion has been used in patients with atrial fibrillation to prevent stroke, but the techniques and devices have shortcomings. This study aims to validate the safety and feasibility of a novel LAA inversion procedure.

**Methods:** LAA inversion procedures were done in six pigs. Before the procedure and at 8 weeks postoperatively, heart rate, blood pressure, and electrocardiogram (ECG) were recorded. The serum concentration of atrial natriuretic peptide (ANP) was measured. The LAA was observed and measured by transesophageal echocardiogram (TEE) and intracardiac echocardiogram (ICE). At 8 weeks after LAA inversion, the animal was euthanized. The heart was collected for morphology and histology, including hematoxylin-eosin, Masson trichrome, and immunofluorescence staining.

**Results:** TEE and ICE showed that LAA was inverted, and the inversion was maintained during the 8-week study duration. Food intake, body weight gain, heart rate, blood pressure, ECG, and serum ANP level were comparable before and after the procedure. Morphology and histological staining showed that there was no obvious inflammation or thrombus. Tissue remodeling and fibrosis were observed at the LAA inverted site.

**Conclusion:** The inversion of LAA effectively eliminates the dead space of LAA and thus may reduce the risk of embolic stroke. The novel procedure is safe and feasible, but the efficacy in reducing embolization remains to be demonstrated in future studies.

## Introduction

Atrial fibrillation (AF) is the most common sustained arrhythmia, and the incidence and prevalence of AF are increasing globally. Based on data from the Framingham Heart Study (Schnabel et al., 2015), the number of patients with AF increased 3-fold over the last 50 years. In U.S. adults, AF prevalence is projected to increase from 5.2 million in 2010 to 12.1 million cases in 2030 (Colilla et al., 2013).

AF increases the risk of stroke by 4–6 times (Reiffel, 2014; NIH, 2022), accounting for over 20% of acute ischemic strokes in 2014 in the US (Alkhouli et al., 2018). In people over 80 years of age, AF is the direct cause of one in four strokes (NIH, 2022). Furthermore, stroke induced by AF has much higher morbidity and mortality than non-AF-related strokes (Reiffel, 2014; January et al., 2019). In addition, it should be noted that stroke risk attributed to AF may be substantially underestimated as AF is frequently asymptomatic or undetected (Healey et al., 2012; Reiffel, 2014; Virani et al., 2021). Although the risk of thromboembolic events is reduced with oral anticoagulation therapy, it is contraindicated in 7.8% of newly diagnosed AF patients (Kakkar et al., 2013), and only 50%–60% of eligible patients with AF receive it (Molteni and Cimminiello, 2014), not to mention that oral anticoagulants may lead to hemorrhagic complications.

The left atrial appendage (LAA) extends from the left atrium (LA). It creates a side chamber, anatomically prone to clot formation and accumulation in a low-flow state such as in AF. The LAA is the nidus for >90% of thrombus formation in AF (Al-Saady et al., 1999; Holmes et al., 2014), where the rapid contraction of the heart that accompanies AF can initiate the release of emboli and the consequent risk of stroke. Percutaneous and surgical approaches to occlude (Alli et al., 2013; Sahay et al., 2017; Della Rocca et al., 2022) or exclude (Bartus et al., 2013; Tilz et al., 2020; Rhee et al., 2021) the LAA have been used to reduce the risk of thromboembolic events. Current devices, however, have major complications (e.g., perforation, migration, and incomplete closure) and disadvantages (e.g., high-cost and foreign body retention). Device-related thrombus and peri-device leak are major issues with LAA occlusion (Simard et al., 2021; Alkhouli et al., 2022; Dukkipati et al., 2022). There is a need to explore novel approaches addressing some of these limitations.

To overcome the limitations of current techniques and devices, we developed a novel procedure to invert the LAA instead of LAA occlusion or exclusion. We hypothesized that LAA inversion, without leaving any devices behind, can eliminate the dead space inside LAA where thrombus tends to form and thus may lower the incidence and risk of embolic stroke. This study aims to support the safety and feasibility of this novel concept in a swine model.

## Methods

### Animal

Six female domestic swine with approximately 60 kg body weight were purchased from S&S Farms (Ramona, CA). The animals were checked to exclude disease after arrival. They were housed at the California Medical Innovation Institute (Calmi^2^) vivarium under constant room temperature and humidity as well as a controlled light-dark cycle. Before undergoing procedures, the animals had at least 5 days for acclimation. Normal food and *ad libitum* access to water were given. All animal experiments were performed in accordance with national and local ethical guidelines, including the Principles of Laboratory Animal Care and the Guide for the Care and Use of Laboratory Animals. The protocol was approved by Calmi^2^ Institutional Animal Care and Use Committee (protocol # 062 and # 062.1).

### LAA inversion procedure

**FIGURE 1:**
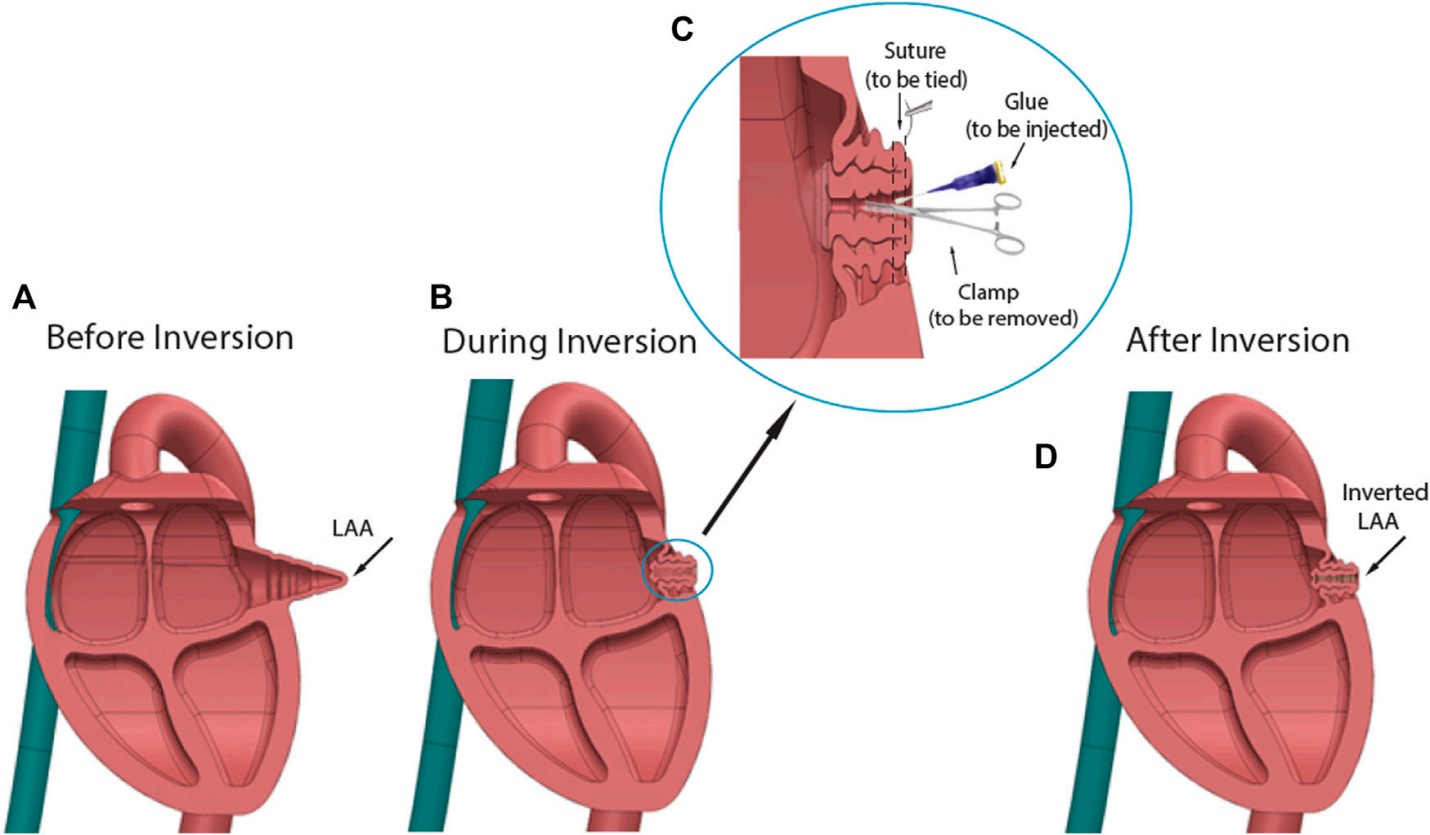
Diagram of LAA inversion procedure. **(A)** Shape of LAA before inversion. **(B)** Using surgical tools and glue to invert the LAA apex. **(C)**. Procedure details: An auricle clamp gently grabbed the LAA apex and put it inward, while tissue glue was applied around the tip of clamp. The purse-string suture was knotted to secure the inversion. **(D)** Shape of inverted LAA. The procedure reduced the dead space in the LAA chamber.

LAA inversion procedure was performed in the six swine following overnight fasting. After sedation with telazol, ketamine, and xylazine, the animal was laid on the operational bed in a supine position with continuous isoflurane inhalation to maintain anesthesia. A transesophageal echocardiogram (TEE) (Philips iE33) was performed for the LAA measurement. Catheters were inserted percutaneously through the jugular vein, femoral artery, and femoral vein, for blood sampling and fluid administration, arterial pressure monitoring, and intracardiac echocardiogram (ICE) (Siemens ACUSON SC 2000). After ICE was completed, the animal was changed to the right lateral position. Following aseptic techniques, an open-chest procedure was performed *via* an intercostal incision (around 15 cm in length). After the exposure of the heart, the LAA was located on the front upper left of the left atrium. The pericardium was then opened, and lidocaine solution was sprayed on the surface of LAA to prevent the arrhythmia from manipulating the heart. A purse-string suture was loosely made around the edge of LAA using 4–0 Prolene (Covidien). An auricle clamp gently grabbed the LAA apex, and the purse-string suture was knotted while putting the LAA apex inward. Initially, tissue glue (Exofin) was used but was found to require reinforcement with several interrupted sutures (4–0 Prolene) to maintain the inversion (see Figure 1 for the diagram of the LAA inversion procedure). The chest was closed layer by layer while negative intrapleural pressure was restored. The intravascular catheters were removed and followed with compressions to stop bleeding. After the procedure, the animal was carefully monitored until complete recovery. Carprofen and buprenorphine were used as analgesics, while the pain assessment score (see Supplementary Material S1) was assessed. Amiodarone oral tablet (200 mg) was given once daily for 7 days to reduce the possibility of acute arrhythmias.

### Intraoperative measurements

During the procedure, body temperature, oxygen saturation, and respiration rate were monitored by an electrocardiogram monitor (Masimo), while electrocardiogram (ECG), heart rate, and arterial blood pressure were monitored and recorded *via* LabChart Pro software (ADInstruments).

TEE was done in a two-chamber longitudinal view for LAA visualization (Yoshimoto et al., 2012). The images were analyzed by ShowCase software (Trillium Technologies). The orifice diameter, depth, and maximum area of the LAA were measured as previously reported (Yoshimoto et al., 2012; Bai et al., 2017; Sarić, 2017). ICE was performed following an established protocol (Baran et al., 2013; Berti et al., 2021).

A terminal measurement was performed at 8 weeks after the LAA inversion procedure. Under general anesthesia, catheters were placed as described above. Heart rate and arterial blood pressure were measured. Blood samples were collected, and imaging (TEE and ICE) was done to determine the geometry of the inverted LAA. The animal was then euthanized by KCL intravenous injection. The heart was harvested for morphological observation and histological studies.

### Blood test

As described above, blood samples were collected at baseline and 8 weeks postoperatively. After centrifugation, the supernatant was collected. An Enzyme-linked immunosorbent assay (Elisa) kit (Reddot Biotech, Canada) was used for testing atrial natriuretic peptide (ANP).

### Histological staining

The inverted LAA was collected and fixed by 4% paraformaldehyde. The right atrial appendage was collected and fixed as a control. Paraffin sections were made at 4 µm. Hematoxylin-eosin (HE), Masson trichrome (MT), and immunofluorescence (IF) staining were performed following standard protocols. In IF staining, anti-fibronectin (Abcam), anti-collagen I (Thermo Fisher Scientific), anti-macrophage (Santa Cruz Biotechnology), and anti-caspase 3 (Abcam) were used as primary antibodies. A digital camera attached to a microscope (Nikon, ECLIPSE E600 or TS2R) was used to observe and photograph the sections.

### Statistical analysis

Quantitative values were expressed as mean ± standard deviation. Using SPSS Statistics software, a t-test was performed for analysis between two groups (baseline vs. termination). *p* < 0.05 was considered statistically significant.

## Results

### Basic information

All LAA inversions were done within 40 min (not including opening and closing the chest). All procedures were performed successfully, and all animals survived until termination. The average body weight of the animals increased from 64.4 ± 4.5 kg (baseline) to 92.7 ± 4.2 kg (termination). The pain assessment score was 4.0 ± 1.5, 1.2 ± 0.8, and 0.3 ± 0.5 on day 1, day 3, and day 5 after surgery, respectively. No severe bleeding, arrhythmia, or other complications happened during and after the procedure. All animals’ food intake, weight gain, and behavior were normal postoperatively. After LAA inversion, no abnormal ECG was observed. Heart rate and blood pressure were slightly higher at 8 weeks compared to baseline, however, all results were in normal range and no statistically significant difference was found (*p* > 0.05), showing stable hemodynamic homeostasis (Table 1).

**TABLE 1:** Hemodynamic parameters. In terms of heart rate, blood pressure, and ejection fraction, no statistically significant difference was found between baseline and termination (*p* > 0.05, t-test) for any of the parameters.

	Baseline	8 Weeks
Heart rate (bpm)	81.9 ± 6.0	89.7 ± 13.5
Mean arterial pressure (mmHg)	62.5 ± 16.5	70.2 ± 15.7
Ejection fraction (%)	57.6 ± 7.9	56.2 ± 4.4

### ANP level

The concentration of serum ANP before the procedure was 482.7 ± 163.5 pg/ml. Eight weeks after LAA inversion, it reached 528.8 ± 193.6 pg/ml. There was no significant difference between these two levels (*p* > 0.05).

### Echo observation

**FIGURE 2:**
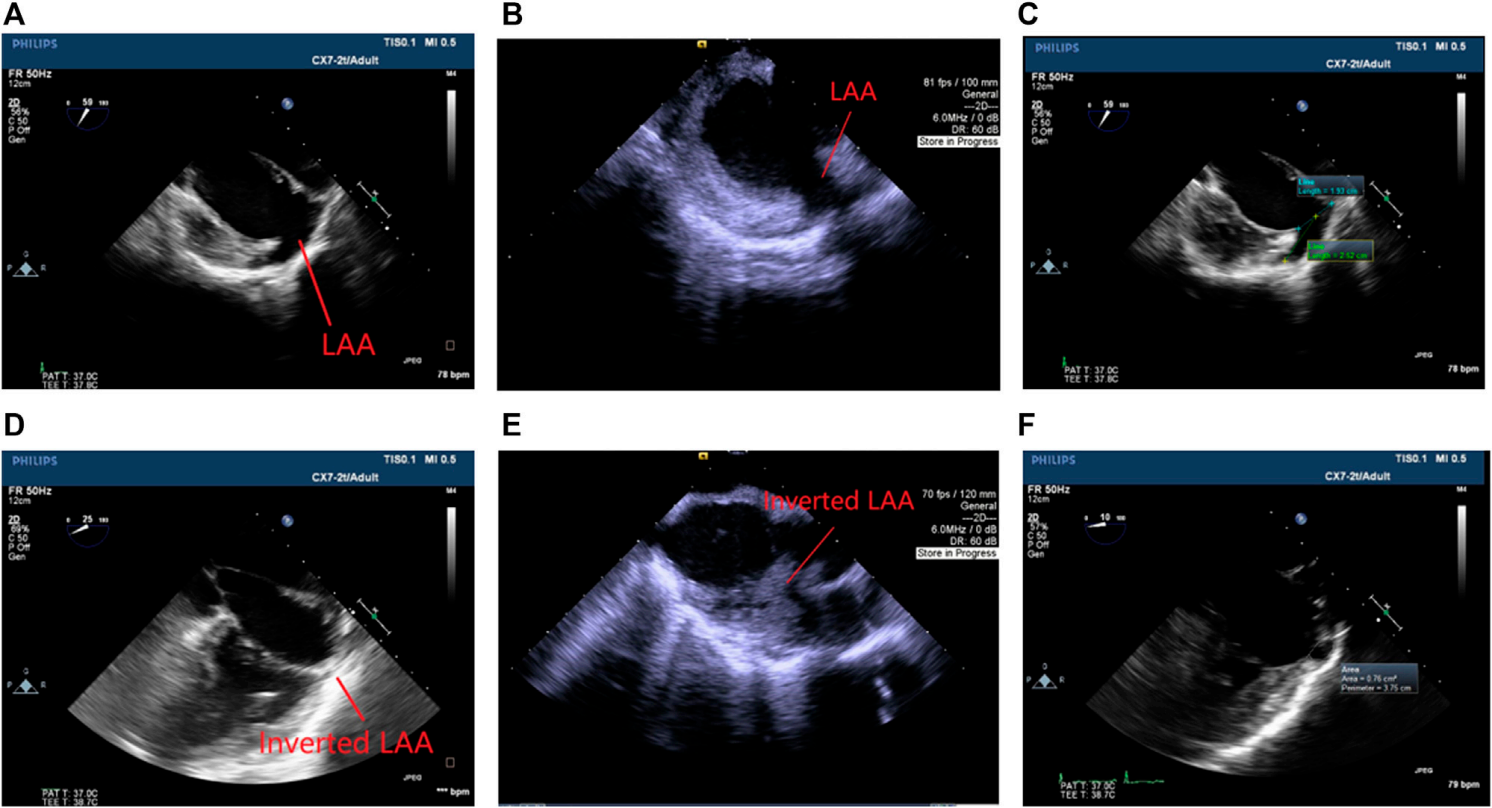
Echocardiogram images. **(A)** TEE before the procedure. **(B)** ICE before the procedure. **(C)** An example of LAA measurement before the procedure (blue: orifice diameter; green: depth). **(D)** TEE at 8 weeks after the procedure. **(E)** ICE at 8 weeks after the procedure. **(F)** An example of LAA measurement at termination (white ring: area). The inversion significantly reduced or eliminated the LAA space.

As recorded by TEE and ICE, the procedure eliminated or significantly reduced the LAA dead space as shown in Figure 2. No thrombus was observed. The ejection fraction was stable. Detailed data in orifice diameter, depth, and maximum area of the LAA or LAA space residue is shown in Table 2.

**TABLE 2:** Dimension of LAA (measured by TEE). After the LAA inversion procedure, the orifice diameter, depth, and maximum area of LAA were all significantly reduced at 8 weeks compared to baseline (*p* < 0.01, t-test). %Change = (8 weeks data–baseline data)/baseline data x 100%.

	Baseline	8 Weeks	%Change	*p-*value
Orifice diameter (cm)	1.7 ± 0.4	1.0 ± 0.2	−40 ± 20%	0.015
Depth (cm)	1.8 ± 0.5	0.9 ± 0.2	−49 ± 5%	0.009
Maximum area (cm^2^)	3.5 ± 0.9	0.7 ± 0.2	−78 ± 4%	<0.001

### Postmortem morphology

**FIGURE 3:**
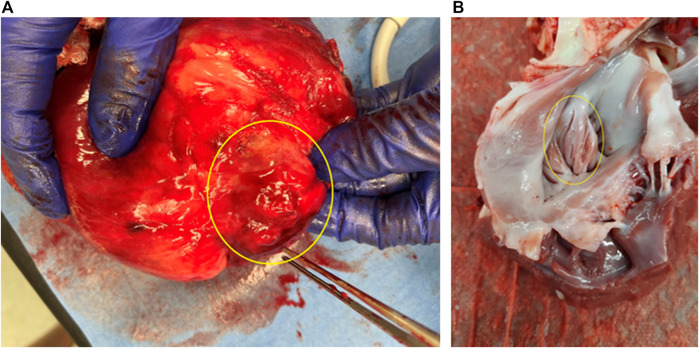
Morphology of LAA inversion (termination at 8 weeks after surgery). **(A)** View from the outside (epicardium). **(B)** View from the inside (endocardium). Yellow rings show the inverted LAA.

After termination, it was found that the inversion was maintained. The inverted tissues had already been wrapped by fibers from the outside, while the inner surface looked normal (Figure 3). No necrosis, damage, or thrombus was observed.

### Histology

**FIGURE 4:**
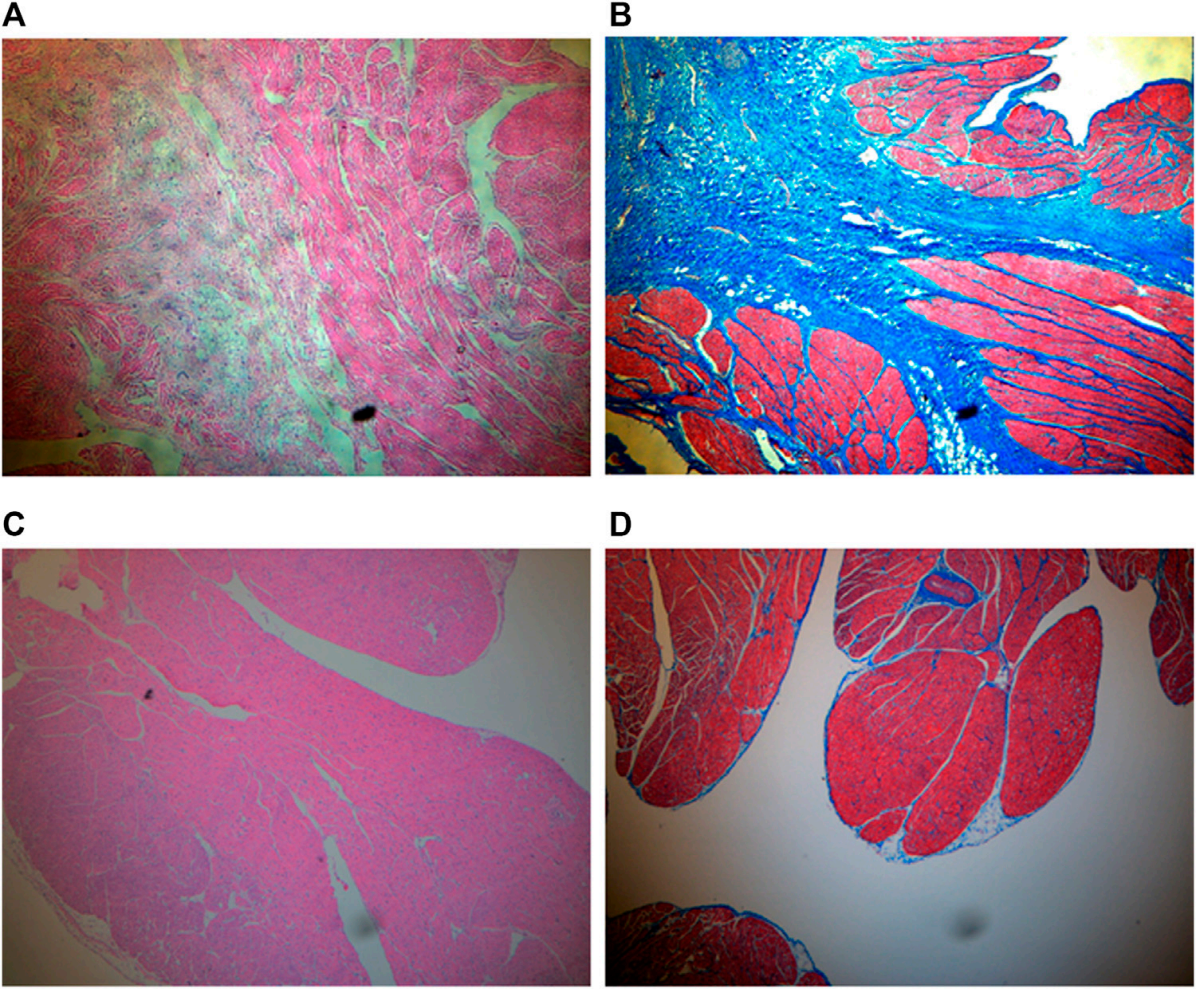
HE and MT staining (×4 objective). **(A)** HE for inverted LAA. Tissue remodeling and fibrosis are shown between myocardia. **(B)** MT for inverted LAA. Collagen expresses in blue, and myocardium expresses in red. **(C)** HE for right atrial appendage as control. **(D)** MT for right atrial appendage as control.

**FIGURE 5:**
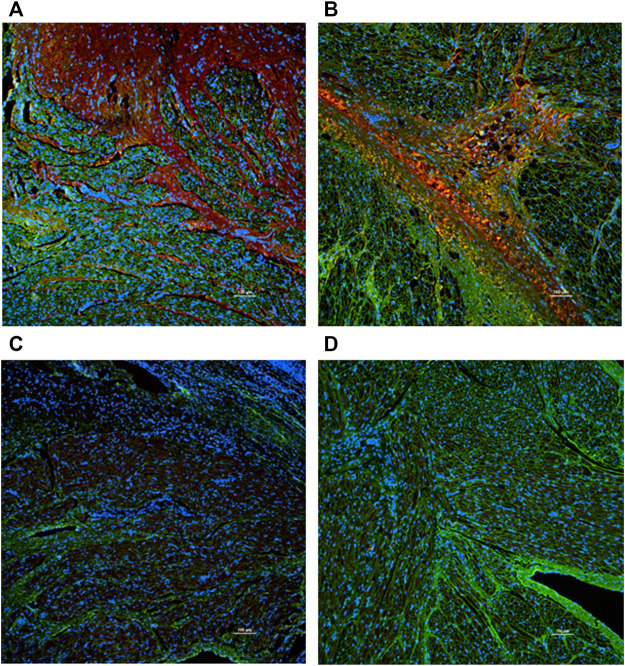
Immunofluorescence staining (×10 objective). Green: Wheat Germ Agglutinin; Blue: Nuclear. Red: Anti-bodies. **(A)** Anti-fibronectin. **(B)** Anti-collagen **(I) (C)** Anti-macrophage. **(D)** Anti-caspase 3. The anti-fibronectin and anti-collagen I were positive (red stain was evident), while the anti-macrophage and anti-caspase were negative (red stain was not present).

HE staining showed that the inverted site exhibited signs of tissue remodeling and fibrosis. No thrombus was shown. MT staining showed that collagen fibers grew significantly among the inverted tissues. These signs were not observed in the right atrial appendage tissues. Images are shown in Figure 4. The fibrosis and collagen growth were also demonstrated by immunofluorescence staining, where anti-fibronectin and anti-collagen I were positively expressed (Figures 5A, B). The anti-macrophage and anti-caspase three were negative (Figures 5C, D), which indicates no apparent inflammation.

## Discussion

We performed the LAA inversion procedure in swine, a good experimental model for cardiovascular procedures, as the heart has a similar size and anatomy compared to humans. The LAA in swine is a triangular structure or shade shaped (Crick, et al., 1998; Ho, et al., 2002). It is broader than humans’ LAA but still has a narrow junction with the rest of the atrium (Ho, et al., 2002), which makes it a good candidate for LAA surgical research. The swine model has been widely used in both LAA procedure evaluation and ischemic stroke studies (Park et al., 2014; Reinthaler et al., 2021; Taha et al., 2022). Using this model, we validated the novel concept of LAA inversion.

Since the LAA is responsible for over 90% of thrombus formation in AF patients, LAA occlusion or exclusion has become a routine treatment (January et al., 2019; Tilz et al., 2020) for reducing the risk of thromboembolic events while negating the need for oral anticoagulation therapy. For example, over 200,000 Watchman implant procedures have been done worldwide (https://www.watchman.com/en-us/how-watchman-device-works.html). Studies also showed that the closure of LAA results in superior thrombotic prevention outcomes than warfarin or antiplatelet therapy (Holmes et al., 2014; Holmes et al., 2015; Sahay et al., 2017). Most current LAA closure procedures, however, must leave a device (e.g., clips (Rhee et al., 2021), Watchman (Friedman et al., 2022), Amplatzer (Ellis, 2022), etc.) in the heart permanently, which may cause migration, LAA perforation, incomplete closure, new thrombus formation, and even thromboembolic events (Gasparini et al., 2012; Reinsch et al., 2016; Dukkipati et al., 2018; Killu et al., 2022; Pracoń et al., 2022). In contrast to these existing approaches, the LAA inversion does not require permanent devices or implants, which is in theory less expensive and less likely to cause adverse events.

Ram et al. (2020) used a similar LAA inversion concept in two patients and achieved good results in a 2-year follow-up. As a matter of record, we introduced the concept of LAA inversion in a patent application published in 2014 (Kassab, 2014), significantly preceding this publication (Ram et al., 2020). Although Ram et al. (2020) performed the procedure based on a vertical left atriotomy, their findings in patients are confirmatory and support the translational value of this approach to patients. Our findings support the merit of LAA resorption without any complications. The pain assessment scoring showed that the animals only experienced mild pain after the LAA inversion procedure (a score <6 was considered mild as shown in the Supplementary Material S1), which was slightly higher at 1-day after surgery and became close to zero (normal) on day 5. These findings indicated that LAA inversion is safe and feasible.

Several procedural issues deserve mention. First, the inversion must be maintained to have successful resorption of the LAA tissue. In the beginning, we found that tissue glue alone was not strong enough to maintain the inversion. On the other hand, sutures provide stabilization of the inversion which lasted for the entire study. To reduce mechanical stimulation of normal heart rhythm during the surgical procedure (Alexander Quinn and Kohl, 2021), lidocaine was dripped on the surface of LAA immediately before the inversion. Pericardial administration of lidocaine increases the thresholds for arrhythmias and ventricular fibrillation (Takada et al., 2007). No obvious cardiac arrhythmia occurred during the procedure or at the time of termination.

The inversion did not affect the heart rate, blood pressure, and ejection fraction, and provided evidence of the procedure’s safety. LAA is considered to have an endocrine and reservoir function (Yamamoto et al., 2022). Therefore, LAA removal or closure may affect LAA’s function, i.e., change the secretion of ANP or blood pressure. Jie et al. (2016) reported that LAA occlusion reduced heart rate and altered ECG, while Liu et al. (2021) stated that these changes returned to the preoperative level in an extended follow-up. Lakkireddy et al. (2018) found that epicardial LAA device implantation lowered blood pressure, while ANP secretion was temporarily inhibited but finally recovered at 3 months postoperatively in these patients. Bartus et al. (2022) reached similar findings. Other studies reported increased (Behnes et al., 2017) or decreased (Luani et al., 2018) ANP levels at 6 months after endocardial LAA closure. We speculate that these variations are mainly due to the different procedure approaches. Unlike these studies, our results showed that the LAA inversion did not change ANP levels or systemic homeostasis including heart rate, blood pressure, and ejection fraction, indicating that LAA inversion is safe and physiological. This difference in finding needs to be further verified in the future.

TEE is the gold standard technology for LAA geometry in patients. Orifice diameter, depth, and area are valid indicators (Yoshimoto et al., 2012; Bai et al., 2017; Freixa et al., 2020) to measure the size of LAA before the device implant and to evaluate residual flow around implanted device after occlusion. After LAA inversion, these parameters were significantly lowered. In traditional LAA closure procedures, residual flow or leakage around the device frequently occurs, although the definition of grading varies in different studies. Sleiman et al. (2021) defined technical success as the absence of a residual leak ≥3 mm, while ≥10 mm was large. So et al. (2021) considered leakage > 5 mm as a major leak. Saw et al. (2017) classified over 5-mm-diameter jet as “severe” and 1–3 mm leakage as “mild”. Whatever the grading is, the incomplete closure of LAA creates a new dead space with a narrower neck and increases the risk of thrombosis and stroke. Even small leaks are associated with increased thromboembolic risks. Previous studies reported that peri-device small leak occurred in above 25% of patients and the incidence of device-related thrombus was 25.3% after LAA occlusion (Simard et al., 2021; Alkhouli et al., 2022; Dukkipati et al., 2022). According to the above definitions, the orifice diameter of the residual space after inversion seemed large (10 mm on average), albeit any device did not occupy the LAA chamber. Along with a shallow cavity (9 mm in depth), the leftover LAA space was small (maximum area 0.7 cm^2^) and hard to form a thrombus. It could potentially have an advantage in mitigating peri-device leak and device-related thrombi. We found no evidence of thrombus by ICE which is effective for detecting thrombus in LAA (Baran et al., 2013) and postmortem observation.

Histological results further validated the safety of the inversion concept at the microstructural level. Commonly, an implanted device may cause mechanical damage or inflammatory injury, but our approach did not induce these adverse effects since there was no foreign body in place. We observed negative expressions of both macrophages, a biomarker for tissue infection and injury (Watanabe et al., 2019; Wang et al., 2021), and caspase 3, an indicator of cell apoptosis (Choudhary et al., 2015; Nichani et al., 2020). These findings suggest that the inversion did not cause inflammation. Furthermore, tissue remodeling and regeneration were demonstrated by fibronectin stain, collagen stain, and MT stain. MT stain is a popular method to identify collagen fibers and differentiate them from muscles (Mao et al., 2016; Otali et al., 2016). This method found an abundance of collagen around the inverted LAA. Subsequently, the proliferation of collagen strengthens the cardiac structural framework and integrates the inverted LAA into surrounding heart tissues. Our group’s recent publication (Pasta et al., 2022), using computational simulation, demonstrated that the inversion of LAA changes the stress distribution from tensile to compressive in the inverted tissue. As shown in the current study, compressive stress results in long-term tissue resorption and hence reduction of the LAA.

### Study limitations

Our study has several limitations. First, we did LAA inversion procedures in normal pigs rather than AF animals. The hemostatic role of the LAA may be more relevant in patients with AF than in those with normal physiology and anatomy. The lack of changes in ANP levels and hemodynamic homeostasis may also be due to a relatively small sample size or because the data was obtained from healthy pigs rather than patients with AF. As creating an AF model (Lin et al., 2003) takes more time, this is a task for future studies. Second, although swine is a popular model in cardiac research, the canine model may be a better model for LAA geometry (Robinson et al., 2018; Wu et al., 2022). We used a swine model because the thoracic cavity is shallower than in canine and hence easier to perform an open-chest procedure. Third, since this study is the first report of LAA inversion in animal models, more examinations and parameters must be established for further assessments. A larger sample size and longer follow-up are also needed. Finally, the use of an open-chest surgery is obviously associated to more disadvantages than a percutaneous procedure. A percutaneous approach to invert LAA must be developed before translating the novel approach to patients.

In conclusion, the concept of LAA inversion is safe and feasible, as demonstrated in the swine model. The inversion of LAA effectively eliminates the dead space of LAA and thus may reduce the incidence of cardiac thrombus. This approach has the potential for AF treatment to prevent embolic stroke, possibly contributing to mitigating a serious health risk for a large population of susceptible patients. There is a potential risk that the LAA inversion procedure may release the clot that is already contained in the LAA chamber. Therefore, before doing the LAA inversion procedure in AF patients, the assessment of thrombus in LAA chamber should be performed. If thrombus already exists, either aspiration of the clot or effective antithrombotic therapies should be used before the procedure.

## Data Availability

The original contributions presented in the study are included in the article/Supplementary Material, further inquiries can be directed to the corresponding author.
