# Supplementary material for: Safety and feasibility of left atrial appendage inversion in swine: A proof-of-concept study for potential therapy to prevent embolic stroke
Source: Front Bioeng Biotechnol. 2023 Feb 16;11:1011121. doi: 10.3389/fbioe.2023.1011121 (PMC9978740; doi:10.3389/fbioe.2023.1011121)
Supplement: Supplementary file 1 [file DataSheet1.PDF]

CALIFORNIA MEDICAL INNOVATIONS INSTITUTE

Pain Assessment Record

PI ____________________ Animal ID ______________________________ Study # __________

Procedure Performed ____

Administer pain medication as stated on the protocol. Continue pain medication if animal has a pain score of score of > 6 out of 24. Notify attending veterinarian for questions or changes.

Date						
Time						
Look at the animal in the cage, is the animal?						
Quiet- 0						
Crying or whimpering, responds to calm voice & Stroking-1						
Groaning, does not respond to calm voice & stroking- 2						
Continuous noise that is unusual for the animal- 3						
Ignoring any wound or painful area- 0						
Looking at wound or painful area- 1						
Licking wound or painful area- 2						
Rubbing wound or painful area- 3						
Chewing wound or painful area- 4						
If it has a wound or painful area, apply gentle pressure 2in around the site. Does it?						
Do nothing- 0						
Look around- 1						
Flinch- 2						
Growl- 3						
Snap- 4						
Cry- 5						
Overall, is the animal?						
Happy & content- 0						
Quiet- 1						
Indifferent or non- responsive to surroundings- 2						
Nervous or anxious or fearful- 3						
Depressed or non- responsive to stimulation- 4						
Comfortable- 0						
Unsettled- 1						
Restless- 2						
Hunched or tensed- 3						
Rigid- 4						
When the animal rises & walks, is it?						
Normal- 0						
Lame- 1						
Slow or reluctant- 2						
Stiff- 3						
It refuses to move- 4						
Total score						
Initials						
